# Research progress on NAT10-mediated acetylation in normal development and disease

**DOI:** 10.3389/fcell.2025.1623276

**Published:** 2025-08-13

**Authors:** Da Qin, Qing Liu, Xiaochao Ma, Rui Wang, Tianyu Lu, Yue Yang, Ze Tang, Yanbo Zhu

**Affiliations:** ^1^ Department of Thoracic Surgery II, Organ Transplantation Center, The First Hospital of Jilin University, Changchun, China; ^2^ Key Laboratory of Organ Regeneration and Transplantation of Ministry of Education, Stem Cell and Cancer Center, The First Hospital of Jilin University, Changchun, China

**Keywords:** NAT10, AC4C, RNA modification, cancer, therapeutic target, epitranscriptomics

## Abstract

N4-acetylcytidine (ac4C) is an evolutionarily conserved RNA modification catalyzed by the acetyltransferase NAT10. It regulates RNA stability, translation, and post-transcriptional processes. Meanwhile, NAT10 functions as a dual-function enzyme exhibiting both protein acetyltransferase and RNA acetylase activities. This review summarizes the structural and functional roles of NAT10-mediated acetylation in physiological contexts, including cell division, differentiation, inflammation, aging, and viral infection, as well as its emerging roles in cancer. In malignancies, NAT10-mediated acetylation drives tumor progression by enhancing mRNA stability, regulating cell cycle, promoting metastasis, suppressing ferroptosis, modulating metabolism, influencing p53 activity, mediating immune escape and fostering drug resistance. Interactions between NAT10 and non-coding RNAs further amplify its oncogenic effects. Unresolved questions, such as microbiota-mediated ac4C regulation and NAT10’s impact on the tumor immune microenvironment, highlight future research directions. Targeting NAT10 and ac4C modification presents promising therapeutic opportunities, with advanced technologies like single-cell sequencing poised to refine epitranscriptome-based interventions.

## Introduction

Classical genetics, rooted in Mendelian laws, elucidates the molecular basis of trait inheritance across generations by investigating gene sequence mutations, recombination patterns, and hereditary principles (Gayon, 2016). However, the completion of genome projects revealed limitations in explaining complex biological phenomena—such as developmental differentiation and environmental responses—solely through DNA sequence variations, prompting the emergence of epigenetics. Epigenetic research focuses on heritable molecular modifications, including DNA methylation, histone modifications, non-coding RNA regulation, and RNA modifications. These modifications influence phenotypic expression without altering genetic sequences by modulating chromatin architecture, gene transcription activity, and RNA metabolism processes (Li, 2021). In the field of tumor biology, aberrant epigenetic regulation has been identified as one of the key drivers of cancer initiation and progression (Farsetti et al., 2023). Within the multi-layered regulatory network of epigenetics, RNA modifications, as critical components of post-transcriptional regulation, have attracted substantial attention in recent years due to breakthroughs in high-throughput sequencing technologies (Sun et al., 2023). Current epitranscriptomic studies have identified approximately 170 types of RNA chemical modifications, with particular emphasis on elucidating the roles of methylation in embryonic development and immune responses. Notably, the acetylated modification N4-acetylcytidine (ac4C), characterized by its unique chemical properties and broad biological effects, is rapidly emerging as a new focal point in epitranscriptomic research (Gilbert and Nachtergaele, 2023).

The acetylation ac4C modification is an evolutionarily conserved RNA epigenetic modification, characterized by the addition of an acetyl group to the N4 position of cytosine, and plays a critical role in mRNA stability and translation (Sas-Chen et al., 2020; Jin et al., 2020). Initially identified in the anticodon loop of tRNA and structural domains of rRNA, this modification was proposed to maintain ribosomal function by stabilizing RNA tertiary structures (Boccaletto et al., 2022). With advancements in mass spectrometry and acetylated RNA immunoprecipitation sequencing (acRIP-seq) technologies, studies have revealed that ac4C exhibits significant enrichment at the 5′ end of mRNA open reading frames (ORFs), suggesting its potential involvement in the regulation of translation initiation (Arango et al., 2018). NAT10, a eukaryotic RNA acetyltransferase, serves as the sole known writer protein for ac4C (Ito et al., 2014). By catalyzing ac4C modification, NAT10 modulates mRNA stability and translation efficiency, thereby participating in biological processes such as cell cycle checkpoint regulation, apoptosis, and DNA damage repair (Dominissini and Rechavi, 2018). Simultaneously, NAT10 functions as a dual-function enzyme exhibiting both protein acetyltransferase and RNA acetylase activities. It catalyzes acetylation of lysine residues in proteins to regulate cell cycle progression and cancer development. In addition to its critical functions in maintaining normal cellular activities and senescence (Wang et al., 2023; Lv et al., 2003), NAT10 has emerged as a key contributor to the development and advancement of multiple malignancies.

The current work provides a systematic framework for understanding the acetylome dynamics governed by NAT10, including their mechanistic underpinnings and cellular consequences. First, it elucidates the catalytic mechanism of NAT10 from a structural biology perspective and summarizes the ac4C modification detection methods. Subsequently, it analyzes the dual roles of NAT10-mediated ac4C modifications in tRNA/rRNA structural maintenance and mRNA metabolic regulation. Further, it explores the physiological functions of NAT10-mediated acetylation in embryonic development and tissue homeostasis. Next, it summarizes the involvement of NAT10-mediated acetylation in non-cancerous diseases. Ultimately, it focuses on its cancer-associated regulatory networks, providing novel insights for developing epitranscriptome-targeted therapies. Given the close association between dynamic ac4C modifications and tumor heterogeneity, future studies should integrate single-cell sequencing with chemical probe technologies to construct spatiotemporally resolved ac4C modification maps. This strategy establishes innovative frameworks for molecularly-targeted diagnostics and therapeutic interventions in next-generation personalized oncology.

## Structure and cellular localization of NAT10

**FIGURE 1:**
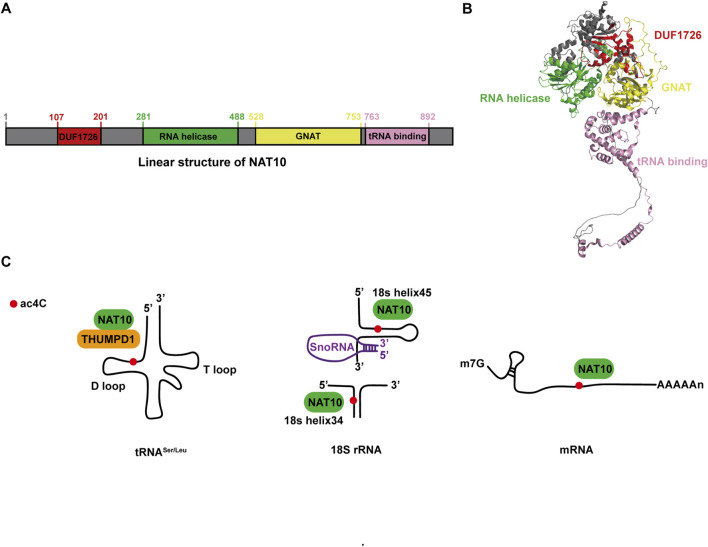
NAT10: Structural characteristics and molecular roles. **(A)** Linear representation of the NAT10 protein. **(B)** Three-dimensional structure of NAT10 obtained from AlphaFold (ID: AF-Q9H0A0-F1), with protein domains visually distinguished using PyMOL. **(C)** Functional overview of NAT10 in tRNA, rRNA, and mRNA.

NAT10 remains the only identified enzyme (writer) responsible for ac4C modification, while its corresponding deacetylases (erasers) and recognition proteins (readers) within the regulatory network are yet to be fully elucidated (Jiang et al., 2023). The NAT10 gene is positioned on chromosome 11 and covers approximately 45 kb in length (Yang et al., 2021a). The NAT10 protein, classified as a histone acetyltransferase within the GCN5-related N-terminal acetyltransferase family, consists of 1,025 amino acids and has a molecular weight of around 116 kDa (Xie et al., 2023a). Structurally, this protein features three primary domains as shown in Figures 1A,B: an acetyltransferase domain (GNAT), a tRNA-binding domain (tRNA binding), and an RNA-release domain (RNA helicase) (Sleiman and Dragon, 2019; Thomas et al., 2019). Acetyl-CoA acts as the essential donor of acetyl groups during ac4C modification, while ATP/GTP hydrolysis supplies the energy required for this enzymatic process (Ikeuchi et al., 2008).

During embryonic organogenesis, NAT10 exhibits a multi-tissue co-expression pattern, with its active regions localized to critical developmental sites including the lymphatic system, hepatic/renal primordia, and the central nervous system (particularly the cerebellar cortex and axonal microenvironment) (Wang et al., 2023). Under normal physiological conditions, NAT10 is predominantly nuclear-localized (Tan et al., 2018). However, during malignant transformation, it undergoes subcellular translocation and mediates pro-tumorigenic effects by activating nucleocytoplasmic transport pathways. In hepatocellular carcinoma (HCC), the intact nuclear localization signals (NLSs) of NAT10 span residues 68–75 and 989–1018. Mutations in these dual NLS fragments result in complete nucleolar exclusion and redistribution of NAT10 to the cytoplasm and plasma membrane. Cytoplasmic NAT10 co-localizes with α-tubulin, while membrane-associated NAT10 interacts with integrins, collectively promoting HCC migration and invasion (Tan et al., 2018). Mutations within nuclear localization signals (NLS) may disrupt NAT10’s compartmentalization in hepatoma cells; however, the mutation probability of NAT10’s NLS in HCC remains unexplored. Future investigations should leverage high-throughput sequencing profiling to delineate specific mutational patterns within NAT10’s NLS domains in HCC. Such insights could guide targeted therapeutic strategies designed to modulate these mutational patterns, reprogram NAT10 distribution, and thereby inhibit cancer progression.Similarly, in colorectal cancer, aberrant subcellular localization of NAT10 is observed, driven primarily by inhibition of glycogen synthase kinase 3β (GSK-3β). This redistribution alters cytoskeletal dynamics, enhancing cancer cell motility (Zhang et al., 2014).

## Summary of ac4C site detection methods and future directions

Detection of ac4C sites in RNA involves several methods, each with its unique advantages and challenges. AcRIP-seq utilizes ac4C-specific antibodies to enrich modified RNA fragments for sequencing, providing broad coverage but at a resolution of 100–200 bp. RedaC:T, a chemical reduction method, offers single-nucleotide resolution by inducing C>T mismatches during reverse transcription. A recent study (Georgeson and Schwartz, 2024) in Molecular Cell reassessed RedaC:T data and concluded that previously reported ac4C sites in human mRNA were not reproducible, attributing them to technical biases. However, this method represents only one approach for detecting ac4C modifications. Multiple studies (Arango et al., 2018; Jiang et al., 2024; Sun et al., 2025) have demonstrated that acRIP-seq using anti-ac4C-specific antibodies identifies thousands of ac4C modification sites in mammalian mRNAs. Meanwhile, this protocol involves NaCNBH_3_ treatment of RNA. Arango et al. (2022) modified the chemical reagents used in ac4C-seq by substituting NaBH_4_ for NaCNBH_3_. This alternative approach detected a similar magnitude of ac4C sites as acRIP-seq, reaffirming the existence of multiple ac4C modification sites in human mRNA. Additionally, computational prediction tools like PACES employ machine learning to predict ac4C sites based on sequence features, offering a rapid screen but requiring experimental validation (Zhao et al., 2019).

To achieve more precise ac4C site detection, integrating orthogonal experimental approaches is crucial. Improved chemical reduction protocols with higher specificity and reduced off-target effects could enhance the reliability of sequencing-based methods. CRISPR-based technologies that allow programmable editing of ac4C sites could provide tools for functional validation. Additionally, enhanced computational models incorporating more comprehensive sequence and structural features may improve prediction accuracy. Ultimately, a multi-faceted approach combining experimental and computational strategies will likely lead to the most robust ac4C site detection and functional characterization.

## Acetylation modification ac4C in diverse RNA species

Gene expression is tightly regulated, progressing from nucleotide sequences to functional proteins, with RNA modifications serving as a potent post-transcriptional regulatory mechanism (Gilbert et al., 2016). Among these, ac4C, a highly conserved post-transcriptional modification involving cytosine acetylation at the N4 position, has emerged as a critical player (Sas-Chen et al., 2020; Yu et al., 2025). Initially thought to be restricted to rRNA and tRNA, ac4C was shown to facilitate rRNA processing via NAT10-mediated acetylation (Ito et al., 2014) and enhance tRNA tertiary structure folding and codon recognition during protein synthesis. Recent advances, however, have revealed its presence in mRNA, expanding its functional repertoire across RNA species (Arango et al., 2018). Below, we summarize the roles of ac4C in these three RNA types (Figure 1C).

tRNA: Current research identifies bacterial TmcA and yeast Kre33 as homologs of NAT10, all belonging to the conserved RNA acetyltransferase family. The ac4C modification was first identified at the wobble position of tRNA^Met^ in *Escherichia coli*. Cytidine acetyltransferase (TmcA) facilitates the formation of ac4C through the action of acetyl-CoA and ATP. This process is essential for ensuring accurate translation by preventing errors in reading isoleucine codons during protein synthesis and for maintaining the stability of the tRNA’s tertiary structure (Ikeuchi et al., 2008). In yeast, the acetyltransferase Kre33, interacting with the conserved adaptor Tan1, was shown to catalyze ac4C modification at the C12 position of tRNA^Leu^ and tRNA^Ser^, ensuring translational accuracy (Sharma et al., 2015). Mechanistically, NAT10 facilitates ac4C deposition by binding to the D-arm of tRNA^Leu^ and tRNA^Ser^, while the THUMP domain of THUMPD1 recruits NAT10 to enhance catalytic efficiency. These ac4C-modified sites in tRNA enhance translational fidelity and sustain organismal thermotolerance, underscoring their essential role in maintaining proteome integrity under stress conditions (Thalalla et al., 2021; Orita et al., 2019).

rRNA: Specific nucleotide modifications in rRNA influence translational accuracy or regulate ribosome biogenesis. For instance, NAT10 mediates ac4C deposition at nucleotide 1842 of mammalian 18S rRNA, a modification critical for ribosome maturation (Ito et al., 2014). In *Schizomyces* sp. And human colorectal cancer HCT116 cells, 18S rRNA harbors two ac4C sites: one located in helix 34 and another in helix 45, both of which contribute to maintaining translational fidelity (Sharma et al., 2015; Bortolin-Cavaillé et al., 2022). Genetic knockout of *NAT10* leads to substantial accumulation of the 30S precursor of 18S rRNA, resulting in growth retardation in human cells. Furthermore, NAT10 interacts with U3 small nucleolar RNA (snoRNA) and acetylates upstream binding factors to activate rRNA transcription, thereby coupling ribosomal RNA synthesis with acetylation-dependent quality control (Kong et al., 2011).

mRNA: Historically, ac4C research focused on tRNA and rRNA, but recent advances have shifted toward its regulatory roles in mRNA. Bioinformatics analyses of ac4C-enriched peaks reveal a striking enrichment of cytidine residues at wobble positions in mRNA transcripts (Arango et al., 2022). Notably, mRNAs with high ac4C content exhibit extended half-lives, highlighting the role of this modification in stabilizing transcripts. Moreover, ac4C in the 5′untranslated region (UTR) directly impacts translation dynamics. For example, ac4C deposition within the Kozak sequence of the 5′-UTR competitively inhibits translation initiation (Arango et al., 2022). These findings underscore ac4C as a dual-functional modification, balancing mRNA stability and translational efficiency.

## The role of NAT10-mediated acetylation in normal cells

**FIGURE 2:**
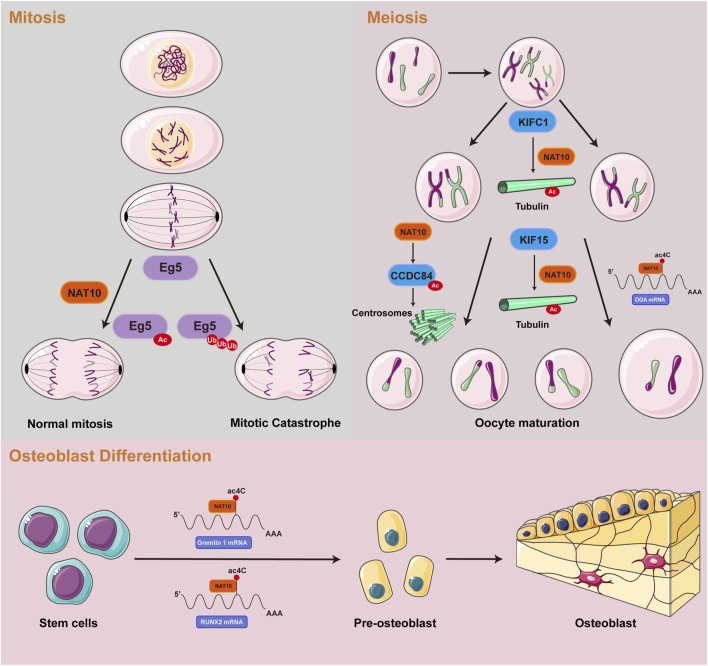
Summary of the roles of NAT10-mediated RNA and protein acetylation in normal cells.

Cellular growth and development encompass tightly regulated processes of proliferation and differentiation. The ac4C RNA acetylation catalyzed by NAT10 promotes the stable expression of genes essential for cell division and differentiation, thus facilitating cellular growth. Furthermore, acetylated tubulins catalyzed by NAT10 not only provide structural support but also facilitate spindle assembly, ensuring accurate chromosome segregation during mitosis and meiosis. This part delineates the functional significance of NAT10-mediated RNA and protein acetylation in normal cellular growth and development, focusing on three key aspects: mitotic processes, meiotic division, and osteoblast differentiation (Figure 2).

## NAT10-mediated acetylation promotes mitosis

Mitosis, a crucial stage of the eukaryotic cell cycle, depends on the precise spatiotemporal control of dynamic microtubule networks. Spindle microtubules, serving as molecular tracks for chromosome segregation, exhibit assembly dynamics strictly controlled by α/β-tubulin heterodimers (Lera-Ramirez et al., 2022; Meunier and Vernos, 2012). Emerging evidence suggests that ac4C may target tubulin mRNAs, potentially influencing spindle assembly and chromosome traction. Genetic ablation or mutation of NAT10 has been shown to induce chromosomal misalignment and segregation errors, leading to multinucleated giant cell formation and mitotic catastrophe (Oh et al., 2017). Complementary studies reveal that NAT10 predominantly localizes to nucleoli during interphase and accumulates at the midbody during late mitosis. Notably, loss of NAT10 function disrupts nucleolar assembly mechanisms and impairs cytokinesis, accompanied by significant reduction in tubulin acetylation levels. During mitotic progression, NAT10 interacts with and co-localizes to kinetochores through E.g.,5 binding. Acetylation of E.g.,5 by NAT10 modulates mitotic cell fate decisions. NAT10 depletion diminishes E.g.,5 loading at kinetochores and compromises their poleward movement, ultimately resulting in monopolar and asymmetric spindle formation (Zheng et al. 2022a). These molecular aberrations may induce cell cycle arrest at G2/M phase or cause temporally delayed mitotic progression through spindle dysfunction, collectively contributing to proliferative defects at the cellular level (Shen et al., 2009).

## NAT10-mediated acetylation promotes meiosis

The meiotic division of mammalian oocytes represents a specialized asymmetric process dependent on microtubule-driven chromosome segregation (Cianfrocco et al., 2015). In mouse oocytes, genetic ablation of the kinesin motor protein KIFC1 disrupts NAT10-mediated tubulin acetylation, resulting in failure of first polar body extrusion (Shan et al., 2022). Concurrently, KIF15 exhibits stage-specific expression during oocyte maturation and colocalizes with microtubules. Functional analyses demonstrate that KIF15 depletion does not alter spindle morphology but induces chromosomal misalignment. Mechanistically, proteomic and co-immunoprecipitation analyses revealed that KIF15 recruits HDAC6, NAT10, and SIRT2 to sustain acetylated tubulin levels, thereby modulating microtubule stability (Zou et al., 2022). Centrosomes, as core regulators of animal cell division, orchestrate spindle microtubule networks to ensure faithful chromosome segregation (Brito et al., 2012). Their numerical integrity, governed by cell cycle kinases, is essential for genomic stability. Recent research has revealed that acetylation of CCDC84 at Lys31 is a dynamic modification regulated collaboratively by the deacetylase SIRT1 and the acetyltransferase NAT10. Fluctuations in CCDC84 acetylation across the cell cycle influence centrosome duplication licensing, thereby impacting meiotic fidelity (Wang et al., 2019). The epitranscriptomic role of ac4C modification emerges as critical during oocyte maturation. Transcriptomic profiling of NAT10-depleted oocytes identified O-GlcNAcase (OGA) as a key ac4C-modified target. NAT10 likely stabilizes OGA by inhibiting its proteasomal degradation, a mechanism essential for oocyte maturation (Lin et al., 2022). RNA pull-down assays conducted in HEK293T cells identified TBL3 as a potential ac4C-binding protein involved in the ac4C modification-dependent regulation of oocyte maturation (Xiang et al., 2021).

Emerging evidence highlights the regulatory significance of ac4C acetylation in mammalian spermatogenesis. Studies have identified dynamic ac4C modifications in mouse testicular mRNAs, with spatiotemporal patterns correlating with key stages of germ cell differentiation. Genetic ablation of NAT10 in germ cells severely suppresses meiotic initiation, causing defects in homologous chromosome pairing, meiotic recombination, and DNA double-strand break repair (Chen et al., 2022). Furthermore, NAT10 depletion reduces global ac4C mRNA levels, generating translationally inactive transcripts that impair meiotic progression and culminate in spermatogenic arrest (Slaidina and Lehmann, 2014).

## NAT10-mediated acetylation drives osteoblast differentiation

Mesenchymal stem cells (MSCs) have the ability to differentiate into multiple lineages and possess significant tissue repair potential (Han et al., 2022). In mice, amniotic fluid-derived MSCs promote corneal cryoinjury repair by activating the ETV4/JUN/CCND2 signaling axis, where ac4C modification enhances mRNA stability of key factors (Fei et al., 2021). In human MSCs, NAT10 increases ac4C levels on Gremlin 1 mRNA, accelerating its degradation to positively regulate osteogenic differentiation (Zhu et al., 2021). In a similar manner, NAT10 promotes osteogenesis in human periodontal ligament stem cells by regulating the VEGFA-PI3K/AKT pathway and ac4C epitranscriptional modifications (Cui et al., 2023). Bone marrow MSCs further demonstrate that NAT10-mediated ac4C deposition on RUNX2 mRNA enhances osteoblast differentiation (Yang et al., 2021b). Altered expression of NAT10 could play a role in the development of osteoporosis, underscoring its potential as a therapeutic target for bone-related diseases.

## The role of NAT10-mediated acetylation in non-neoplastic diseases

Acetylation modifications influence inflammatory factor release, telomerase activity, and viral infection efficiency. Thus, we summarize the specific roles of NAT10-mediated acetylation modifications in inflammatory diseases, aging-related diseases, and viral infections.

## NAT10-mediated acetylation regulates inflammatory response

Sepsis, a severe condition triggered by pathogenic infections, is a major cause of mortality in intensive care units. Recent research has highlighted the role of pyroptosis in the progression of sepsis (Kaukonen et al., 2014). Zhang et al., 2022a). Demonstrated that specific overexpression of NAT10 in neutrophils significantly suppresses pyroptosis, thereby improving survival rates and alleviating lung injury in septic mice. Mechanistically, reduced NAT10 expression accelerates ULK1 mRNA degradation, leading to upregulated expression of the pro-pyroptotic NLRP3 inflammasome in neutrophils, providing a potential therapeutic target for sepsis. The study analyzing differences in urine composition between interstitial cystitis patients and healthy subjects via mass spectrometry found significantly elevated ac4C modification levels in the urine of interstitial cystitis patients. Furthermore, it revealed a significant negative correlation between ac4C modification levels and the expression of uromodulin, a key protein in urinary tract resistance to bacterial infection (Parsons et al., 2014). Systemic lupus erythematosus (SLE) is a long-term autoimmune disorder marked by the dysregulated activation of the immune system, leading to attacks on the body’s own tissues, causing widespread inflammation and damage to multiple organ systems. Guo et al. (2020). Analyzed 11 RNA modification levels in CD4^+^ T cells of SLE patients and observed a significant reduction in ac4C modification on mRNAs. Using acRIP-seq, they mapped transcriptome-wide ac4C modification profiles in SLE CD4^+^ T cells, uncovering molecular mechanisms by which ac4C regulates the stability of key target mRNAs (e.g., USP18, GPX1, and RGL1) and protein synthesis initiation to participate in disease pathogenesis.

## NAT10-mediated acetylation promotes aging

Hutchinson-Gilford progeria syndrome (HGPS) is a rare, untreatable disorder characterized by accelerated aging. Understanding the disrupted biological mechanisms in HGPS could pave the way for the development of new therapeutic approaches. The study investigated the pathogenic role of NAT10-mediated acetylation of tubulin in HGPS (Larrieu et al., 2018). It revealed that upregulated NAT10 acetylates tubulin, enhancing its stability and increasing its binding affinity for TNPO1, blocking nuclear entry and molecular transport, ultimately inducing transcriptional silencing in HGPS cells. Remodelin, a NAT10-targeted inhibitor, reduces tubulin acetylation to release TNPO1. The released TNPO1 transports cargo protein Nup153 into the nucleus, which binds to LaminsA at nuclear pores to form the nuclear pore complex basket structure. Finally, TNPO1 delivers hnRNPA1 into the nucleus to restore transcriptional activity and correct HGPS cellular phenotypes, offering potential therapeutic strategies for HGPS and normal aging-related disorders. Alzheimer’s disease (AD) is a neurodegenerative condition marked by the accumulation of amyloid plaques in the brain. This study investigated the relationship between ac4C modifications and AD, along with the mechanisms through which ac4C contributes to the progression of the disease (Ma et al., 2022a). Analyses using acRIP-seq and RNA-seq on AD mice and their wild-type counterparts identified notable differences in the abundance of ac4C modifications in lncRNAs, offering a basis for further investigation into the regulatory processes involved (Ma et al., 2022a).

## NAT10-mediated acetylation modulates viral infection and replication

Multiple studies indicate that ac4C modifications enhance viral replication capacity and pathogenicity by increasing viral RNA protein synthesis rates and nucleic acid structural stability (Furuse, 2021; Courtney, 2021). In a transcriptome study, NAT10 was found to directly interact with multiple influenza virus components, including Polymerase Basic Protein 1 (PB1), Nucleoprotein (NP), Neuraminidase (NA), and Matrix Protein 1 (M1) proteins. Knockdown of NAT10 negatively regulates viral growth, suggesting its potential pro-viral role (Watanabe et al., 2014). The HIV-1 Tat protein, a viral-encoded trans-activator, is crucial for the regulation of viral transcription. Proteomic studies have shown a significant interaction between NAT10 and Tat (Jean et al., 2017). NAT10 protein suppresses Tat-mediated HIV-1 transcription, indicating its involvement in maintaining HIV-1 latency. Thus, NAT10 represents a potential pharmacological target for further investigation in “shock and kill” HIV-1 cure strategies. Hao et al. (2022). Elucidated the biological significance of ac4C modifications in enterovirus 71 (EV71) replication. They demonstrated that NAT10 mediates ac4C modifications in the 5′UTR of the EV71 genome, and inhibition of NAT10 activity significantly suppresses EV71 replication. EV71 strains with markedly reduced ac4C acetylation exhibit attenuated pathogenicity in mouse models. Mechanistically, ac4C modifications enhance viral RNA translation efficiency by specifically recruiting PCBP2 protein to the internal ribosome entry site regulatory element. These findings establish critical molecular targets for developing antiviral drugs based on RNA epigenetic regulation.

## The role of NAT10-mediated acetylation in tumors

**FIGURE 3:**
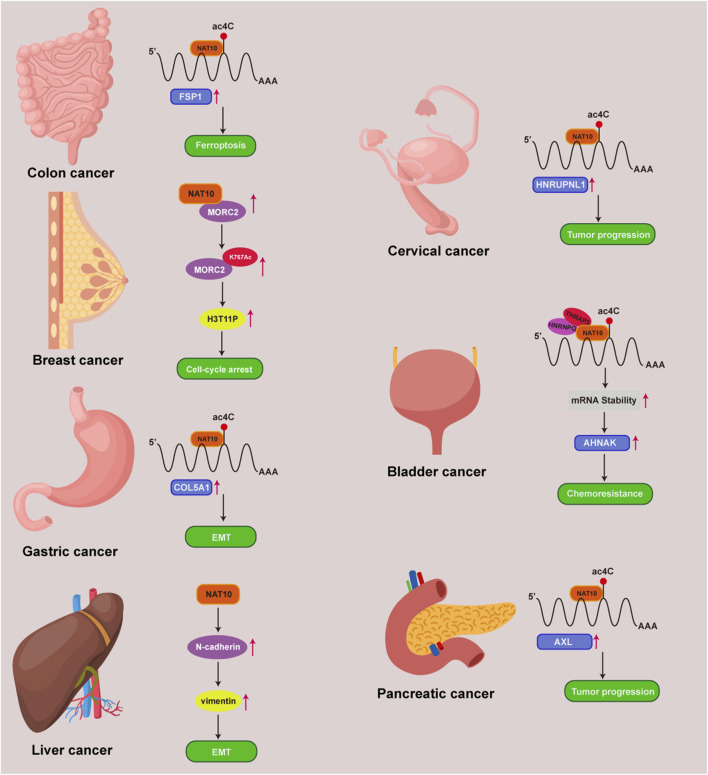
NAT10 and ac4C mechanisms in multiple tumors. Mechanisms of NAT10 and ac4C modification in colon, breast, gastric, liver, cervical, bladder, and pancreatic cancers.

Cancer represents a major threat to human health, and its development is closely linked to ac4C modifications (Ouyang et al., 2024; Zhang et al., 2024). NAT10 specifically catalyzes ac4C epigenetic modifications on oncogenic RNAs and acetylation on proteins in tumor tissues, driving malignant progression. Regulating RNA modifications has become a potential therapeutic approach for cancer, with NAT10-mediated ac4C modulation identified as a promising target for cancer treatment. However, the molecular mechanisms of NAT10 in tumorigenesis remain incompletely elucidated, and its functional characterization remains a frontier area. We have therefore conducted a retrospective analysis of NAT10’s role in cancer, systematically integrating its cancer regulatory network to reveal its translational potential as a novel therapeutic target (Figure 3) (Table 1). We will comprehensively elaborate on NAT10’s tumor-related functions from nine perspectives: improving mRNA stability, regulating cell cycle, promoting tumor metastasis, suppressing ferroptosis, interfering with metabolic pathways, modulating p53 activity, mediating immune escape, conferring drug resistance, and non-coding RNA-mediated regulation of NAT10.

**TABLE 1:** NAT10-mediated mechanisms across cancer types.

Cancer	Expression	Mechanisms	Downstream gene or pathway	Ref
Melanoma	Up	Cell cycle S phase arrest	MITF	Oh et al. (2017)
Multiple myeloma	Up	Improving mRNA stability	BCL-XL、CEP170、PI3K-AKT pathway	Wei et al. (2022), Zhang et al. (2022b)
Osteosarcoma	Up	Improving mRNA stability	FNTB	Zhang et al. (2023a)
Acute myeloid leukemia	Up	Inducing cell apoptosis	UPR pathway	Zi et al. (2020)
Squamous cell carcinoma of the head and neck	Up	Cell cycle S/G2 phase arrest	-	Tao et al. (2021)
Esophagus cancer	Up	Improving mRNA stability	NOTCH3	Liao et al. (2023)
	Up	Activating EMT	lncRNA CTC-490G23.2	Yu et al. (2023)
	Up	Improving mRNA translation efficiency	EGFR	Wei et al. (2023)
Breast cancer	Up	Suppressing ferroptosis	GCLC, SLC7A11	Dalhat et al. (2023)
	Up	Enhancing drug resistance	PARP1, MORC2	Qi et al. (2022)
Gastric cancer	Up	Activating EMT	COL5A1	Zhang et al. (2021)
	Up	Inducing glycolysis	SEPT9 HIF-1 pathway	Yang et al. (2023a)
	Up	Improving mRNA stability	Mdm2/p53 pathway	Deng et al. (2023)
Pancreatic cancer	Up	Improving mRNA stability	AXL	Zong et al. (2023)
Hepatocellular carcinoma	Up	Improving mRNA stability	Mutant p53	Li et al. (2017)
	Up	Enhancing drug resistance	HSP90AA1	Pan et al. (2023)
Colorectal cancer	Up	Affecting DNA replication	SASP pathway	Cao et al. (2020)
	Up	Suppressing ferroptosis	FSP1	Zheng et al. (2022b)
	Up	Promoting tumor metastasis	KIF23, Wnt/β-catenin pathway	Jin et al. (2022)
	Down	Improving mRNA stability	P53	Liu et al. (2016)
Bladder cancer	Up	Improving mRNA stability	BCL9L, SOX4, AKT1	Wang et al. (2022a)
Prostatic cancer	Up	Affecting DNA replication	CDC6	Ma et al. (2022b)
	Up	Activating EMT	KRT8	Li et al. (2024)
Cervical cancer	Up	Improving mRNA stability	HNRNPUL1	Long et al. (2023)

## Improving mRNA stability

**FIGURE 4:**
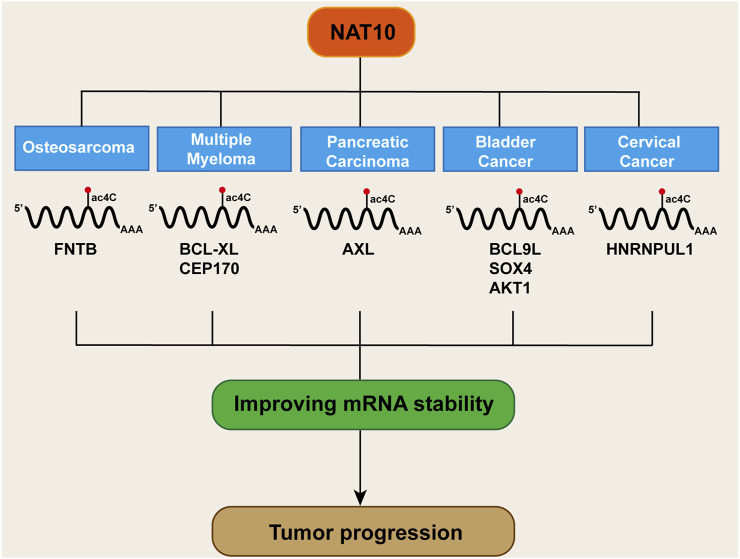
Mechanisms of NAT10 and ac4C in enhancing mRNA stability in cancer.

The occurrence of tumors is governed by the RNA homeostasis regulatory network, involving transcript stability, RNA-protein interactions, and translation fidelity (Liu et al., 2022a; Lin et al., 2024). NAT10 mediates ac4C modification of mRNA to delay degradation while regulating codon decoding accuracy to enhance translational efficiency. This dual regulatory mechanism leads NAT10 to exhibit critical oncogenic phenotypes across various cancer types (Figure 4).

In osteosarcoma, acRIP-seq analysis identified the farnesyltransferase subunit beta gene (FNTB) as a target gene undergoing ac4C acetylation. In osteosarcoma cells, treatment with the acetyltransferase inhibitor Remodelin led to a decrease in FNTB mRNA stability and protein translation efficiency (Zhang et al., 2023a). AcRIP-seq analysis revealed BCL-XL as a downstream target of NAT10 in multiple myeloma. Further mRNA stability assays showed that NAT10 stabilizes BCL-XL mRNA and enhances protein translation, thereby preventing apoptosis (Zhang et al., 2022b). Similarly, in multiple myeloma, acRIP-seq combined with ribosome profiling sequencing (Ribo-seq) confirmed CEP170 as a critical downstream target of NAT10. Overexpression of CEP170 was found to promote cell proliferation and chromosomal instability in multiple myeloma (Wei et al., 2022). In pancreatic cancer, RNA-seq screening identified the receptor tyrosine kinase AXL as a downstream target of NAT10. AcRIP-qPCR and mRNA stability assays confirmed that NAT10 boosts AXL mRNA stability in an ac4C-dependent manner, resulting in elevated AXL expression, which in turn promotes the proliferation and metastasis of pancreatic cancer cells (Zong et al., 2023). In bladder cancer, NAT10 knockdown specifically attenuated mRNA ac4C modification, impairing translation efficiency of BCL9L/SOX4/AKT1 and accelerating degradation of BCL9L/SOX4 transcripts (Wang et al., 2022a). Studies in cervical cancer also demonstrated that NAT10 enhances HNRNPUL1 mRNA stability through ac4C modification, thereby promoting cancer cell proliferation, invasion, and migration (Long et al., 2023). Current evidence confirms that NAT10 dynamically regulates cancer-associated transcriptome homeostasis via ac4C acetylation to drive malignant tumor phenotypes. This raises critical questions: whether there exists an ac4C deacetylase that reverse-regulates this modification network, and whether NAT10 exerts tumor-suppressive functions through maintaining mRNA structural homeostasis of proto-oncogenes. These inquiries provide novel perspectives for deciphering the bidirectional molecular switch functionality of NAT10.

## Regulating cell cycle

The cell cycle consists of a series of highly regulated processes that ensure the accurate replication of genomic DNA and the formation of two daughter cells (Nurse, 2000). The accuracy and integrity of DNA replication, as well as the faithful segregation of sister chromatids, are rigorously quality-controlled through distinct cell cycle checkpoints (Bertoli et al., 2013). Genetic alterations or dysregulation of cell cycle regulators, along with disrupted signaling at cell cycle checkpoints, can lead to improper cell cycle re-entry and aberrant cell division, which constitute hallmark features of cancer (Malumbres and Barbacid, 2009; Matthews et al., 2022). Consequently, cell cycle regulation represents a rational therapeutic target for anticancer strategies (Da Costa et al., 2023). Studies have shown that reduced NAT10 expression impedes the recruitment of key nucleolar assembly factors, causes defective cleavage furrow ingression, and significantly diminishes tubulin acetylation. These phenotypic changes result in a higher frequency of G2/M phase arrest and extended mitotic exit duration, indicating that NAT10, as an epigenetic regulator, is essential for cell division. It achieves this by preserving the structural continuity between the nucleolus and midbody, as well as ensuring the mechanical stability of microtubules (Shen et al., 2009). Live-cell imaging in another study demonstrated that NAT10 knockdown significantly prolongs mitotic duration and induces chromosome misalignment, indicating its role in safeguarding chromosomal segregation fidelity through epitranscriptomic regulation (Zheng et al., 2022a). These findings emphasize the vital role of NAT10 in cell cycle regulation, highlighting the importance of further investigation to understand its regulatory functions in cancer.

In melanoma, NAT10 silencing induces S-phase cell cycle arrest by downregulating Microphthalmia-associated Transcription Factor (MITF) expression, significantly suppressing tumor cell proliferation in both *in vitro* and *in vivo* experimental systems (Oh et al., 2017). In colorectal cancer, NAT10 facilitates the formation of micronuclei (MN) during DNA replication. NAT10-positive MN triggers the Senescence-associated Secretory Phenotype (SASP) pathway through its interaction with cGAS. Clinical translational studies revealed that NAT10 expression levels exhibit significantly positive correlations with MN generation frequency and SASP pathway activity in colorectal cancer cohorts, with coordinated elevation of these parameters in tumors exhibiting poor differentiation, advanced TNM stages, and high metastatic potential. Mechanistically, NAT10 promotes malignant progression by playing dual roles in micronuclei formation and SASP activation, highlighting its potential as a novel prognostic biomarker and therapeutic target (Cao et al., 2020). In prostate cancer, NAT10 was found to interact with DNA replication complexes and directly bind to cell cycle protein CDC6, participating in DNA replication processes (Ma et al., 2022b). In acute myeloid leukemia, inhibiting NAT10 promotes apoptosis by triggering endoplasmic reticulum stress, which activates the unfolded protein response (UPR) pathway and subsequently initiates the canonical apoptotic pathways (Zi et al., 2020). Studies in lung cancer (Wang et al., 2022b) and head and neck squamous cell carcinoma (Tao et al., 2021) similarly demonstrated that NAT10 knockdown significantly prolongs cell cycle arrest in G1 or S/G2 phases.

## Promoting tumor metastasis

Metastasis is the most dangerous characteristic of malignant tumors, responsible for around 90% of cancer-related deaths due to the spread of cancer cells rather than the primary tumors themselves (GANESH and Massagué, 2021). Despite advances in molecular biology, scientific blind spots persist in metastasis research due to spatiotemporal heterogeneity, the complexity of microenvironmental interactions, and the plasticity of molecular regulatory networks (Fares et al., 2020). The epithelial-mesenchymal transition (EMT), a key driver of tumor metastasis, enhances tumor cell invasiveness, circulatory survival, and distant colonization through epigenetic reprogramming, establishing it as a major focus in anti-metastatic therapy (Yang et al., 2020; Dongre and Weinberg, 2019). EMT is a transdifferentiation process through which transformed epithelial cells acquire invasive, stress-resistant, and disseminative capabilities (Hanahan and Weinberg, 2011; Gu et al., 2024). Recent studies reveal that NAT10 acts as a molecular switch for EMT progression in multiple malignancies via its unique ac4C RNA modification mechanism, while also influencing chemotherapy resistance, offering novel perspectives for precision oncology.

In prostate cancer, NAT10 promotes metastasis by acetylating Keratin 8 (KRT8) mRNA to enhance its stability, downregulating E-cadherin and upregulating N-cadherin protein expression, thereby activating EMT (Li et al., 2024). In gastric cancer, NAT10 directly interacts with the 3′UTR of COL5A1 mRNA, modulating its ac4C modification. This modification extends the half-life of COL5A1 mRNA, leading to increased expression of EMT markers Vimentin and MMP2, which promotes metastasis in gastric cancer (Zhang et al., 2021). In colon cancer, NAT10 stabilizes KIF23 mRNA by binding to its 3′UTR and enhancing ac4C modification. Increased levels of KIF23 protein activate the Wnt/β-catenin pathway, leading to the nuclear translocation of β-catenin and promoting tumor progression and metastasis. Post-translational modifications expand proteomic complexity (Jin et al., 2022). In esophageal cancer, NAT10 is identified as a substrate of 2-hydroxyisobutyrylation (Khib). Khib modification enhances NAT10’s binding affinity with deubiquitinase USP39, significantly prolonging NAT10 protein half-life. Downstream, NAT10 increases NOTCH3 mRNA stability via ac4C acetylation to promote esophageal cancer metastasis (Liao et al., 2023). A study in esophageal squamous cell carcinoma showed that NAT10-mediated ac4C modification induces overexpression of lncRNA CTC-490G23.2 in primary tumors, with even higher levels in metastatic tissues. Mechanistically, lncRNA CTC-490G23.2 serves as a scaffold to facilitate the binding of CD44 pre-mRNA with PTBP1, promoting oncogenic splicing from CD44s to CD44v isoforms. The CD44v isoforms interact with Vimentin, stabilizing its protein and enhancing metastasis (Yu et al., 2023). NAT10 similarly facilitates metastasis in cervical cancer (Chen et al. 2024a) and hepatocellular carcinoma (Ma et al., 2016) through regulation of key EMT-related proteins.

Current studies confirm a significant association between EMT and tumor therapy resistance (De Craene and Berx, 2013). Pharmacological evaluations in breast cancer (Wu et al., 2018) and hepatocellular carcinoma (Zhang et al., 2019) models demonstrate that NAT10 silencing effectively counteracts doxorubicin-induced EMT, thereby reversing drug-resistant phenotypes in tumor cells. Consequently, combining NAT10-targeted inhibition with chemotherapeutic agents produces synergistic antitumor effects, reducing metastatic lesions, attenuating chemotherapy resistance, suppressing proliferative activity of metastases, and expanding indications for R0 resection surgery.

## Suppressing ferroptosis

Ferroptosis is a form of regulated cell death induced by disturbances in iron metabolism, characterized by the accumulation of lipid peroxidation products that lead to the disruption of membrane structures. Its molecular mechanisms and pathological significance have recently emerged as a hotspot in oncology research (Ru et al., 2024). Unlike traditional cell death pathways like apoptosis and necroptosis, ferroptosis is characterized by distinct morphological and biochemical characteristics (Jiang et al., 2021; Dixon et al., 2024). For instance, apoptosis is characterized by chromatin condensation, nuclear pore complex disintegration, and double-membrane vesicle formation, whereas ferroptosis specifically manifests mitochondrial matrix densification, asymmetric phospholipid distribution in the outer membrane, and cristae remodeling defects (Stockwell et al., 2017). This death pathway molecularly couples with oxidative damage of polyunsaturated fatty acids (PUFAs) in membrane phospholipid bilayers, regulated by the glutathione peroxidase 4 (GPX4) system, iron homeostasis, and lipid peroxidation repair mechanisms. Mechanistically, ferroptosis initiation is governed by three key pathways: 1) inactivation of the glutathione-GPX4 antioxidant axis leading to impaired lipid peroxide clearance; 2) free iron accumulation due to iron-sulfur cluster biosynthesis defects; 3) lipoxygenase (LOX)-mediated membrane phospholipid peroxidation (Friedmann et al., 2019; Lei et al., 2024). Notably, epigenetic regulation significantly modulates ferroptosis susceptibility. Research has shown that DNA methylation, histone acetylation, and non-coding RNAs modulate tumor cell responses to ferroptosis inducers by regulating key genes such as FSP1, ACSL4, and SLC7A11 (Lee and Roh, 2023).

**FIGURE 5:**
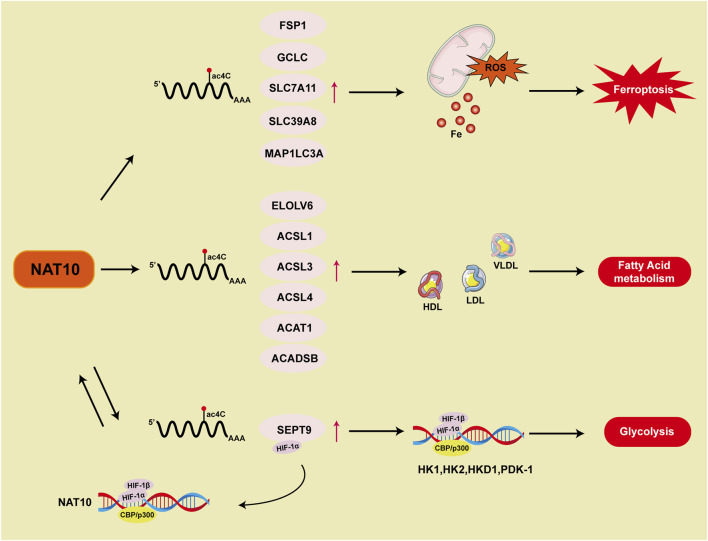
Summary of NAT10 and ac4C in modulating ferroptosis and metabolic mechanisms.

Recent studies reveal that NAT10-catalyzed ac4C epigenetic modification regulates tumor ferroptosis (Figure 5). Ferroptosis suppressor protein 1 (FSP1), a critical negative regulator, establishes a multi-layered protective mechanism: 1) suppressing lipid peroxidation radical amplification; 2) maintaining dynamic stability of membrane phospholipid bilayers; 3) synergizing with glutathione metabolic networks to balance intracellular redox homeostasis (Li et al., 2023). In colon cancer (Zheng et al., 2022b), NAT10 modulates the FSP1 expression axis via ac4C modification, driving a ferroptosis-suppressive microenvironment characterized by reduced ROS, Fe^2+^, Malondialdehyde (MDA), mitochondrial matrix condensation, and cristae reduction, indicating that acetylated ac4C modification of FSP1 mRNA correlates with ferroptosis inhibition. In breast cancer (Dalhat et al., 2023), NAT10 knockout significantly downregulates ferroptosis-essential genes (SLC7A11, GCLC, MAP1LC3A, and SLC39A8). Mechanistically, reduced ac4C levels shorten GCLC and SLC7A11 mRNA half-lives, decreasing cystine uptake and glutathione (GSH) levels while elevating ROS, lipid peroxidation, and oxidized phospholipids, thereby promoting ferroptosis. Current research focuses on ac4C modification of ferroptosis-related genes. Future studies could explore how NAT10 influences tumor ferroptosis through fatty acid metabolism regulation and whether NAT10 reverses drug resistance via ferroptosis pathways.

## Interfering with metabolic pathways

A core hallmark of malignant tumors is their uncontrolled proliferative capacity, which fundamentally differs from normal cellular biological behavior (Zhao et al., 2021). Recent investigations employing comparative genomics, metabolomics, and epigenetics have systematically unveiled dysregulated metabolic networks in cancer cells. Metabolic reprogramming was established as a central cancer hallmark in the updated “Hallmarks of Cancer” proposed by Hanahan and Weinberg in 2011 (Counihan et al., 2018; You et al., 2023). Tumor metabolic reprogramming, marked by the Warburg effect and enhanced anabolic processes, plays a key role in tumor initiation, metastasis, drug resistance, and the upkeep of cancer stem cells (Park et al., 2020). Many well-known oncogenes and tumor suppressor genes contribute to maintaining this altered metabolic state in cancers (Li et al., 2020). Epigenetic modifications and metabolic changes are closely intertwined and regulate each other in cancer progression (Sun et al., 2022).

The tumor hypoxic microenvironment, a pathological hallmark of malignant progression, drives glycolytic metabolic remodeling through the HIF-1α molecular hub (Lee et al., 2020). This suggests that cancer cells predominantly depend on glycolysis rather than mitochondrial oxidative phosphorylation to support vital biological processes (Greene et al., 2022). HIF-1α, a key regulator of the cellular response to hypoxia, accumulates in low oxygen environments and triggers the expression of hypoxia-adaptive genes like hexokinase, lactate dehydrogenase, and pyruvate dehydrogenase kinase, which in turn enhances glycolysis (Keith et al., 2011). As a result, excessive activation of the HIF-1 signaling pathway leads to glycolytic dependence. In gastric cancer, NAT10 stimulates the HIF-1 pathway and alters glucose metabolism through ac4C modification of SEPT9 mRNA. In glycolysis process, HIFs binding to DNA causes transcription activation of NAT10. The NAT10/SEPT9/HIF-1 positive feedback loop further enhances HIF-1 pathway activation, reinforcing the reliance on glycolysis (Yang et al., 2023a) (Figure 5).

Lipid metabolism, which includes lipid synthesis, breakdown, and utilization, is frequently reprogrammed in cancer to support the elevated energy needs and rapid proliferation of malignant cells. This metabolic remodeling represents a cancer hallmark, enabling tumor cells to sustain their proliferative capacity, evade apoptosis, and adapt to the tumor microenvironment (Vogel et al., 2024; Wang et al., 2020). Studies demonstrate that NAT10-mediated ac4C modification is associated with fatty acid metabolism (Figure 5). Mechanistic investigations reveal that fatty acid metabolism-related genes (ELOVL6/ACSL1/ACSL3/ACSL4/ACADSB/ACAT1) are regulated through NAT10-dependent ac4C epitranscriptomic modifications, which stabilize their mRNA. Genes involved in fatty acid metabolism modulate serum levels of high-density lipoprotein (HDL), low-density lipoprotein (LDL), and very low-density lipoprotein (VLDL), thereby influencing systemic metabolic homeostasis. Genetic intervention confirms that NAT10 depletion induces compensatory reductions in total lipid pools, triglycerides, and cholesterol levels (Dalhat et al., 2022). Further research using untargeted metabolomics analysis through high-performance liquid chromatography-tandem mass spectrometry (HPLC-MS/MS) in Remodelin-treated cancer cells shows changes in mitochondrial fatty acid metabolism and lipid accumulation (Dalhat et al., 2021).

## Modulating p53 activity

The tumor suppressor gene p53, as one of the most frequently mutated genes in cancer, plays a central role in tumorigenesis and progression due to its dysfunction (Hassin and Oren, 2023). Wild-type p53 suppresses cancer cell formation through multiple mechanisms, including regulation of the cell cycle, DNA repair, and apoptosis. Mutations or inactivation of p53 are critical factors driving the initiation and progression of numerous cancers, making the restoration of p53 function a key research direction in cancer therapy (Bykov et al., 2018). Mdm2, a core regulatory hub for p53, is closely associated with p53 protein expression. Mdm2 specifically recognizes the transcriptional activation domain of p53 and forms a complex with it, initiating an E3 ubiquitin ligase-dependent ubiquitination cascade that directs p53 to the 26S proteasome degradation pathway (Prives, 1998). This regulation is part of a negative feedback mechanism: when p53 is activated (e.g., under DNA damage or other stress conditions), it promotes Mdm2 expression by activating transcriptional responses. In turn, the expressed Mdm2 suppresses p53 activity by facilitating its degradation, thereby maintaining intracellular p53 homeostasis (Liu et al., 2024).

Numerous studies indicate that NAT10 regulates p53 expression to influence tumor progression. In gastric cancer, NAT10 catalyzes ac4C modification of Mdm2 transcripts, driving imbalance in the Mdm2/p53 regulatory axis by maintaining mRNA epitranscriptomic homeostasis, thereby promoting gastric carcinogenesis. Additionally, *Helicobacter pylori* infection upregulates NAT10 expression, leading to Mdm2 overexpression and subsequent p53 degradation. Further research demonstrates that targeting NAT10 with Remodelin exhibits anticancer activity in gastric cancer and enhances the antitumor efficacy of Mdm2 inhibitors in p53 wild-type gastric cancer (Deng et al., 2023). In hepatocellular carcinoma, NAT10 enhances the stability of mutant p53, increasing its expression and promoting cancer progression (Li et al., 2017). However, other studies reveal that NAT10 catalyzes site-specific post-translational modification of p53 at the K120 site, bypassing Mdm2-mediated ubiquitination degradation to stabilize p53. Furthermore, NAT10 activates E3 ligase-dependent ubiquitin-proteasome degradation of Mdm2, reducing Mdm2 expression to suppress cell proliferation (Liu et al., 2016). These conflicting findings may arise from the following factors: First, the mutational status of p53 (wild-type vs. mutant) in different tumor cells directly determines the functional direction of NAT10. Second, cell type-specific post-transcriptional regulatory networks (e.g., differential microRNA expression profiles) may alter the interaction patterns between NAT10 and its targets. Additionally, existing studies predominantly rely on *in vitro* cell models, lacking consideration of tumor microenvironmental influences (e.g., immune cell infiltration, hypoxic conditions). For instance, in 3D culture or patient-derived xenograft models, NAT10-mediated regulation of the p53-Mdm2 axis may exhibit more complex spatiotemporal dynamics.

## Mediating immune escape

The tumor immune microenvironment (TME) is a complex and dynamic ecosystem within tumor tissues, consisting of various immune cells, cytokines, and regulatory networks, and plays a crucial role in controlling tumor development and progression (Kubli et al., 2021). Key abnormalities in the TME include an overabundance of immunosuppressive cells such as regulatory T cells and tumor-associated macrophages, altered expression of immune checkpoint proteins like PD-1/PD-L1, and disrupted secretion of pro-inflammatory cytokines. Together, these factors contribute to immune escape and support tumor malignancy (Hanahan et al., 2025). Recent progress in immunotherapy, especially immune checkpoint inhibitors (ICIs) and adoptive cell therapies, has transformed the treatment of cancers. However, clinical outcomes indicate that only 20%–30% of patients experience long-lasting remission, highlighting the urgent need to better understand the molecular mechanisms regulating the tumor immune microenvironment (TME) to enhance therapeutic effectiveness (Kennedy and Salama, 2020). Epigenomic features in both immune cells and cancer cells may serve as predictive biomarkers for immunotherapy outcomes (Hogg et al., 2020; Yang et al., 2023b). At the same time, potential epigenetic biomarkers may offer a foundation for patient stratification and personalized treatment approaches, optimizing therapeutic outcomes while reducing side effects (Villanueva et al., 2020).

In-depth analyses utilizing The Cancer Genome Atlas (TCGA) and Genotype-Tissue Expression (GTEx) data demonstrated notable positive correlations between NAT10 expression and components of immune infiltration (B cells, CD8^+^ T cells, CD4^+^ T cells, neutrophils, macrophages, dendritic cells, endothelial cells, and fibroblasts) in hepatocellular carcinoma (Yang et al., 2021a). Another study in hepatocellular carcinoma developed an ac4C Score model, demonstrating that high-score tumors exhibited advanced staging, higher p53 mutation rates, elevated tumor stemness, increased immune scores, and heightened regulatory T cell infiltration, suggesting the ac4C Score as a novel prognostic indicator for anti-PD1 immunotherapy response (Liu et al., 2022b). In pancreatic cancer, a molecular subtyping system with prognostic value was constructed by integrating NAT10-regulated gene expression profiles through bioinformatics analysis of TCGA pancreatic cancer cohorts. Results indicated that the subtype with the poorest prognosis showed extensive immune cell infiltration and activated interferon-γ(IFN-γ) signaling pathways, implying potential heightened responsiveness to immune checkpoint inhibitors compared to other subtypes (Xu et al., 2023). In colorectal cancer, a novel prognostic prediction model was established using multi-omics data integration of acetylation-associated differential genes. This risk scoring system exhibited significant positive correlations with dynamic immune cell infiltration levels, microsatellite instability (MSI) phenotypes, tumor mutational burden (TMB), and sensitivity to immune checkpoint inhibitor therapy (Zhang et al., 2023b). Although the specific role of NAT10 in immunotherapy remains unclear, investigating whether NAT10 targeting could enhance immunotherapy sensitivity and elucidating mechanisms by which NAT10 regulates immunity via ac4C modifications holds critical significance.

## Conferring drug resistance

In cancer treatment, the evolution of acquired drug resistance mechanisms constitutes a major clinical challenge, directly driving relapse and distant metastasis events (Jin et al., 2023). Recent studies indicate that aberrant epitranscriptomic modifications can remodel the tumor microenvironment through multidimensional regulatory networks, endowing cancer cells with the ability to evade therapeutic pressure (Lu et al., 2020; Mabe et al., 2024). As a result, targeting RNA modification regulators offers a promising approach to overcome therapeutic resistance and improve treatment effectiveness (Chen et al., 2024b).

In breast cancer, NAT10 overexpression mediates acetylation modifications of PARP1 to regulate its protein homeostasis. This post-translational modification event triggers remodeling of the DNA damage response pathway, facilitating efficient recruitment of repair complexes at γH2AX-marked sites, ultimately inducing adaptive resistance to platinum-based chemotherapy by maintaining survival advantages in cancer stem cells (Qi et al., 2022). In another breast cancer study, NAT10 promotes DNA damage-induced G2 checkpoint activation via acetylation of MORC2 at lysine 767. This epigenetic regulatory mechanism drives cross-resistance to genotoxic agents (gemcitabine/cisplatin) and radiotherapy. Preclinical models confirmed that chemical inhibition or depletion of NAT10 induces replication stress response defects, forcing cells to bypass checkpoint barriers and restoring chemosensitivity through synthetic lethality effects (Liu et al., 2020). In bladder cancer, cisplatin activates NF-κB signaling, which increases NAT10 transcription, resulting in enhanced mRNA stability through ac4C modification and improved DNA damage repair, thus promoting chemoresistance. When NAT10 is inhibited pharmacologically with Remodelin alongside cisplatin, the efficacy of chemotherapy is significantly enhanced (Xie et al., 2023b).

NAT10 not only enhances resistance to chemotherapy and radiotherapy but also holds significance in targeted therapies. In hepatocellular carcinoma, NAT10 activates the endoplasmic reticulum stress axis while concurrently upregulating ac4C modification levels of HSP90AA1 mRNA, thereby increasing HSP90AA1 protein expression. This dual regulatory mechanism promotes invadopodia formation linked to the EMT process in hepatocellular carcinoma cells and induces resistance to the tyrosine kinase inhibitor Lenvatinib (Pan et al., 2023). In esophageal cancer, NAT10-mediated ac4C modification of tRNA boosts the translational efficiency of Epidermal Growth Factor Receptor (EGFR) mRNA. The combined depletion of NAT10 and treatment with the EGFR small-molecule inhibitor gefitinib synergistically inhibits esophageal cancer progression both *in vitro* and *in vivo* (Wei et al., 2023).

## Non-coding RNA-mediated regulation of NAT10

During malignant tumor progression, non-coding RNAs (ncRNAs) drive coordinated regulation of proteome expression by forming competing endogenous RNA (ceRNA) networks (Xiong and Zhang, 2023; Schmitt and Chang, 2016). The hierarchical diversity of this epigenetic regulatory layer has been identified as a core driver of tumor heterogeneity. The diversity of ncRNAs, including microRNAs (miRNAs), long non-coding RNAs (lncRNAs), and circular RNAs (circRNAs), constitutes intricate regulatory networks. For example, lncRNAs can competitively bind miRNAs to relieve their inhibitory effects on target mRNAs, while circRNAs stabilize interactions with miRNAs or RNA-binding proteins through their closed circular structures (Hansen et al., 2011). Simultaneously, ncRNA networks act as master regulators of cellular epigenetic features by modulating critical processes such as histone modifications and DNA methylation, thereby influencing multiple oncogenic pathways (Roy et al., 2023; Chen et al., 2020). Multiple studies suggest that ncRNAs can interact with NAT10, further impacting cancer development.

In gastric cancer, the lncRNA DARS-AS1 acts as a ceRNA by binding to miR-330-3p, which in turn regulates NAT10 expression and promotes cancer progression (Du et al., 2022). In colon cancer, miR-6716-5p interacts with the 3′UTR of NAT10 mRNA, resulting in decreased NAT10 protein levels and affecting the progression of the disease (Liu et al., 2019). In pancreatic cancer research, lncRNA LINC00623 has been shown to bind NAT10 mRNA and recruit the deubiquitinase USP39, preventing its degradation via the ubiquitin-proteasome pathway and consequently elevating NAT10 expression levels (Feng et al., 2022). Although current research predominantly focuses on ncRNA-mediated regulation of NAT10, the functional impact of ac4C modification on ncRNAs themselves remains to be thoroughly explored. As an emerging RNA epigenetic mark, ac4C may influence RNA stability, subcellular localization, and protein-binding capacity by altering RNA secondary structures. In mRNAs, NAT10-mediated ac4C modification has been shown to enhance translational efficiency and prolong half-life, suggesting that NAT10 may exert similar mechanisms in ncRNAs. For instance, ac4C modification of lncRNAs could modulate their binding efficiency to chromatin-modifying complexes or regulate miRNA sponge effects through specific spatial conformations. Furthermore, ac4C modification near circularization sites of circRNAs may influence back-splicing efficiency, thereby regulating their biogenesis and functionality. Therefore, future investigations into the role of ac4C modification in ncRNAs represent a critical frontier in epigenetic research.

## Discussion

**FIGURE 6:**
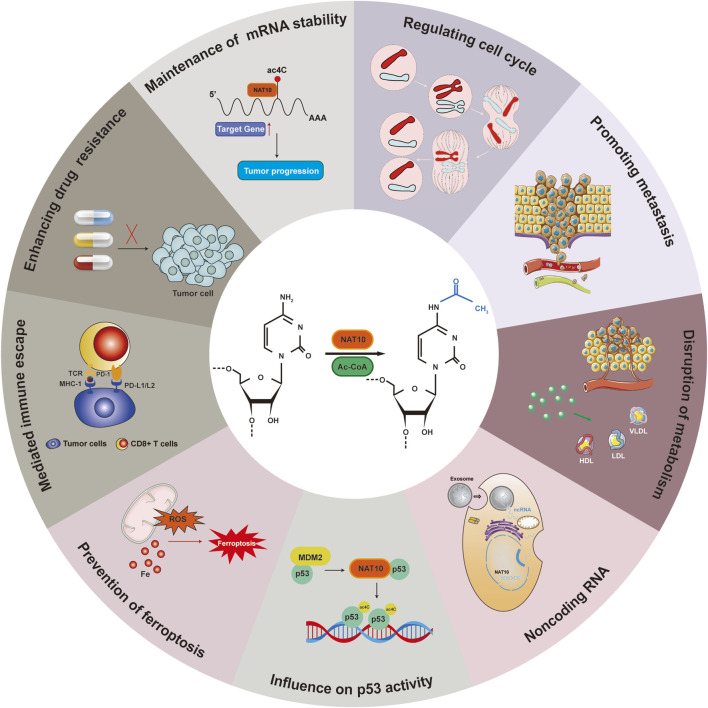
NAT10-mediated acetylation: regulatory mechanisms in cancer.

As an RNA modification, ac4C not only plays critical roles in normal cellular activities but also contributes to disease pathogenesis through diverse mechanisms. The review summarizes nine aspects through which NAT10-mediated acetylation influences cancer progression (Figure 6). However, several questions warrant further investigation, such as the impact of microbiota metabolism on ac4C epigenetic modification, the specific effects of NAT10 on the tumor immune microenvironment, and whether deacetylases exist to counteract NAT10-driven oncogenesis. Additionally, it is imperative to conduct more detailed investigations into the specific molecular pathways altered by NAT10. For instance, modifying its oncogenic effects through site-specific mutations that alter RNA-binding capacity, rather than globally reducing NAT10 expression, could minimize adverse effects in therapeutic applications. Therefore, future research should prioritize translating foundational insights on NAT10 into clinical applications.

## Abbreviations

ac4C, N4-acetylcytidine; acRIP-seq, acetylated RNA immunoprecipitation sequencing; ORFs, Open reading frames; HCC, Hepatocellular carcinoma; NLSs, Nuclear localization signals; GSK-3β, Glycogen synthase kinase 3β; TmcA, Cytidine acetyltransferase; snoRNA, small nucleolar RNA; UTR, Untranslated region; OGA, O-GlcNAcase; MSCs, Mesenchymal stem cells; SLE, Systemic lupus erythematosus; HGPS, Hutchinson-Gilford progeria syndrome; AD, Alzheimer’s disease; EV71, Enterovirus 71; FNTB, Farnesyltransferase subunit beta; Ribo-seq, Ribosome profiling sequencing; MITF, Microphthalmia-associated Transcription Factor; MN, Micronuclei; SASP, Senescence-associated Secretory Phenotype; UPR, Unfolded protein response; EMT, Epithelial-mesenchymal transition; KRT8, Keratin 8; Khib, 2-hydroxyisobutyrylation; PUFAs, Polyunsaturated fatty acids; GPX4, Glutathione peroxidase 4; FSP1, Ferroptosis suppressor protein 1; HPLC-MS/MS, High-performance liquid chromatography-tandem mass spectrometry.
